# P27 Promotes TGF-*β*-Mediated Pulmonary Fibrosis via Interacting with MTORC2

**DOI:** 10.1155/2019/7157861

**Published:** 2019-09-19

**Authors:** Yu-heng Dai, Xiao-qing Li, Da-peng Dong, Hai-bo Gu, Cheng-ying Kong, Zhihao Xu

**Affiliations:** ^1^Hangzhou Women's Hospital, Hangzhou 310008, China; ^2^Second Hospital of Yingzhou District, Ningbo 315040, China; ^3^The First Affiliated Hospital of Wenzhou Medical University, Wenzhou 325000, China; ^4^The Fourth Affiliated Hospital, School of Medicine, Zhejiang University, Yiwu 322000, China

## Abstract

Pulmonary fibrosis (PF), a progressive and life-threatening pulmonary disease, is the main pathological basis of interstitial lung disease (ILD) which includes the idiopathic pulmonary fibrosis (IPF). No effective therapeutic strategy for pulmonary fibrosis has been established. TGF-*β* signaling has emerged as the vital regulator of PF; however, the detailed molecular mechanisms of TGF-*β* in PF were uncertain. In the present study, we proved that inhibition of MTORC2 suppresses the expression of P27 in MRC5 and HLF cells. And in bleomycin-induced PF model, the expression of *α*-SMA and P27 was upregulated. Moreover, TGF-*β* application increased the level of *α*-SMA, vimentin, and P27 in MRC5 and HLF cells. Furthermore, P27 overexpression advanced the cell cycle process and promoted the proliferation of MRC5 and HLF cells. Finally, the rescue experiment showed that MTORC2 knockdown reversed P27 overexpression-induced cell cycle acceleration and proliferation. Thus, our results suggest that P27 is involved in TGF-β-mediated PF, which was regulated by MTORC2, providing a novel insight into the development of PF.

## 1. Introduction

Pulmonary fibrosis is a lung disease that is hard to cure and leads to a high mortality rate. It includes a variety of lung diseases characterized by the progressive and irreversible destruction of lung architecture caused by scar formation that ultimately leads to organ malfunction, disruption of gas exchange, and death from respiratory failure [1]. Idiopathic pulmonary fibrosis (IPF) is the most common and severe pulmonary fibrotic disorder, identified as a progressive and lethal pulmonary disease with unknown etiology and uncertain pathogenesis [2]. The morbidity of IPF is about 3 to 9 cases per 100,000 person-years [3]. The prognosis of IPF patients is poor, with the average survival time being only 2–4 years. However, the clinical outcomes of IPF vary widely, and some patients can survive for a long time [4]. Despite the latest progress of antifibrosis treatment, IPF is still an incurable disease [5]. There is still much work to be done to explore novel targets and develop strategies and/or biomarkers to reverse pulmonary fibrogenesis.

Previous literature demonstrates that frequently occurring enriched myofibroblasts with *α*-smooth muscle actin (*α*-SMA) expression are the fibroblastic foci of IPF lung tissue [6]. Locations of activated lung fibroblasts are frequently overlapped with *α*-SMA-positive myofibroblasts [7, 8]. Activated lung fibroblasts secrete ECM (extracellular matix) components, such as collagen and laminin [9]. TGF-*β* can effectively promote the differentiation of fibroblasts into myofibroblasts and increase the secretion of ECM components [10]. In addition, TGF-*β* promotes tissue remodeling and cell-ECM interactions via inducing the expression of integrins, matrix metalloproteinases, protease inhibitors, and regulators of small GTPases [11]. Meanwhile, secreted by macrophages and metaplastic alveolar epithelial cells, TGF-*β* was found to be upregulated in IPF lung tissue [12, 13]. TGF-*β* was also proved to induce epithelial-mesenchymal transition (EMT). Previous literature revealed that TGF-*β*-induced EMT in alveolar epithelial cells may partly contribute to the change of epithelial characters in IPF lung tissue [14]. Thus, it is conceivable that TGF-*β* may contribute to IPF pathogenesis. Further experiments are required to determine the exact mechanism of TGF-*β* in the fibrotic process of IPF.

Studies of mTORC2 complex are less abundant than that of the mTORC1 complex, but its role in cell proliferation and survival was well described. mTORC2 consists of several components, including the rapamycin-insensitive companion of mTOR (Rictor), mLST8/G*β*L, mammalian stress-activated protein kinase-interacting protein 1 (mSIN1), Protor 1/2, DEPTOR, TTI1, and TEL2 [15, 16]. Recent evidence demonstrated that mTORC2 may be related to pulmonary fibrosis via a TGF-*β*-dependent pathway [17, 18]. TGF-*β* was reported to upregulate Rictor in fibroblasts of IPF lung tissue, activating mTORC2 and AKT pathways [18]. Pathological AKT activity of fibroblasts through abnormal activation of mTORC2 signaling is feasible, which helps to induce antiapoptosis phenotype of the fibroblasts in IPF focal fibrous tissue. However, how mTORC2 functions in TGF-*β*-mediated PF diseases including IPF is still unknown.

In the present study, we aimed to explore the detailed mechanisms of TGF-*β*-mediated PF. We found that mTORC2 could promote TGF-*β*-mediated PF via regulating P27 expression.

## 2. Method and Materials

### 2.1. Cell Culture and Transfection

Human fetal lung fibroblasts, MRC5, and HLF were gained from the American Type Culture Collection (ATCC, Manassas, VA), cultured in minimal essential medium (MEM) (Gibco, USA) with 10% (v/v) FBS and 1% penicillin/streptomycin. For cell transfection, the P27 and Rictor siRNA were synthesized by GenePharma (Shanghai, China), and P27 overexpressed vector and control blank vector were gained from OriGene (Rockville, MD, USA). Before the tansfection, cells were cultured to 60%–80% confluence and transfected using Lipofectamine 2000 (Invitrogen, CA, USA) according to the manufacturer's instructions.

### 2.2. Western Blotting

The cells were collected and lysed, and sample proteins were quantitated using pierce BCA protein assay (Thermo Fisher Scientific, Rockford, USA). Equal amounts of proteins were separated by SDS-PAGE and transferred to PVDF membranes. Primary antibodies and dilutions used for Western blotting included the following: anti-P27 (1 : 1,000 dilution, Abcam, Boston, MA, USA), antivimentin (1 : 1000 dilution, Abcam), and mouse anti-GAPDH (1 : 4,000 dilution, Abcam); anti-*α*-SMA (1 : 1,000 dilution, Abcam), anti-*β*-actin (1 : 2,000 dilution, Cell Signaling, Beverly, MA, USA), anti-MTORC-p (1 : 1000 dilution, Abcam), and anti-Rictor (1 : 2000 dilution, Abcam). Secondary antibody (horseradish peroxidase-conjugated immuno-pure anti-IgG; H + L) was used at a dilution of 1 : 5,000. The blots were developed with chemiluminescence reagent ECL kit (Beit Haemek, Israel).

### 2.3. Mouse Lung Fibrosis Model

The animal procedures were approved by the Institutional Animal Care and Use Committee at Zhejiang University. SPF C57BL/6 male mice (6–8 weeks old) were adopted and divided into two groups: normal saline group and bleomycin injection group (2.5 U/kg). Under anesthesia, the neck skin was incised, and the trachea was exposed. Saline or 2.5 U/kg bleomycin (Sigma, USA) insulin was injected into the mouse trachea from the interval of the trachea cartilaginous rings with a 25-guageneedle. Then, the tracheal puncture points were sutured by silk thread. Mouse lungs were collected at 7, 14, and 28 days for experiments.

### 2.4. Quantitative Real-Time PCR (qRT-PCR)

Total RNA was extracted using the TRIzol reagent (Invitrogen, Carlsbad, CA) according to manufacturer's instructions. Then, reverse transcription was performed to get the first strand cDNA by using the PrimeScript® RT reagent kit (TaKaRa, Dalian, China). The expression level of P27 was determined by qPCR reactions and were performed by using the ABI 7500 Fast system (Applied Biosystems, CA) with SYBR green (TaKaRa). The 2^−ΔΔCt^ method was used for quantification. All reactions were triplicated. The relative expression of P27 was respectively normalized to GAPDH.

### 2.5. Immunohistochemistry

Immunostaining was done on formalin-fixed, paraffin-embedded mice lung tissue specimens, and 4*μ*m thick paraffin sections were cut. These sections were incubated overnight with anti-p27 and anti-*α*-SMA antibody (Abcam) at a 1 : 200 dilution in 5% FBS overnight and then incubated with the horseradish peroxidase-conjugated secondary antibody for 30 min at room temperature. The results were visualized by reaction with diaminobenzidine (DAB; 3,3′-diaminobenzidine tetrahydrochloride) and counterstaining with hematoxylin.

### 2.6. Cell Proliferation Assay

MRC5 and HLF cells were plated at a density of 1.0 × 10^3^ cells/well in 96-well plates. The cells were cultured in the corresponding serum-free medium for 24 h. Cell viability was examined using10 *μ*L/well CCK-8 solution (Dojindo, Kumamoto, Japan). After incubating for 2 h, the absorbance was measured at 450 nm using a MRX II microplate reader (Dynex, Chantilly, VA, USA).

### 2.7. Cell Cycle Assay

MRC5 and HLF cells with different treatments were trypsinized after washing twice with PBS and then fixed at 20°C in 75% ethanol for 12 h. Next, the cells were incubated with 0.5 mL DNA Prep Stain (Beckman Coulter, USA) in the dark at room temperature for 30 min. Then, the cell cycle was determined by the flow cytometry (BD FACScanto II, BD Biosciences, USA).

### 2.8. Statistical Analysis

Results are presented as means ± SEM. Significance of the differences between means was assessed using one-way analysis of variance or a two-tailed Student's *t*-test. Values of *P* less than 0.05 were considered significant.

## 3. Results

### 3.1. P27 Was Overexpressed in Bleomycin-Induced Mouse Lung Fibrosis Model

**Figure 1:**
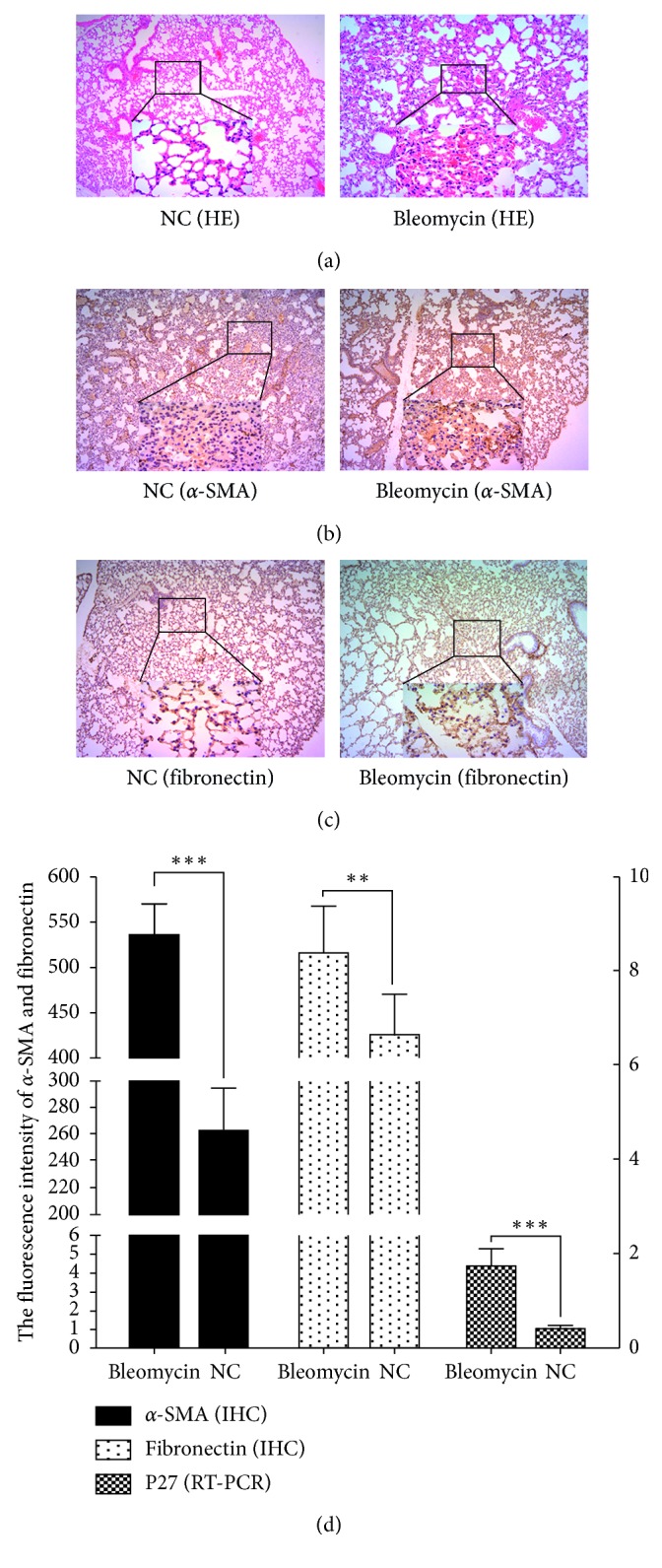
Expression of a-SAM, fibronectin, and P27 in bleomycin mouse lung fibrosis model. (a) H&E staining of lung fibrotic tissue in the NC and the bleomycin group. (b-c) Expression of a-SAM and fibronectin in the NC and the bleomycin group by IHC. (d) The relative expression of a-SAM, fibronectin, and P27 in the NC and the bleomycin group. The results of a-SAM and fibronectin expression were from the IHC staining density, and the results of P27 expression were from RT-PCR.

**Figure 2:**
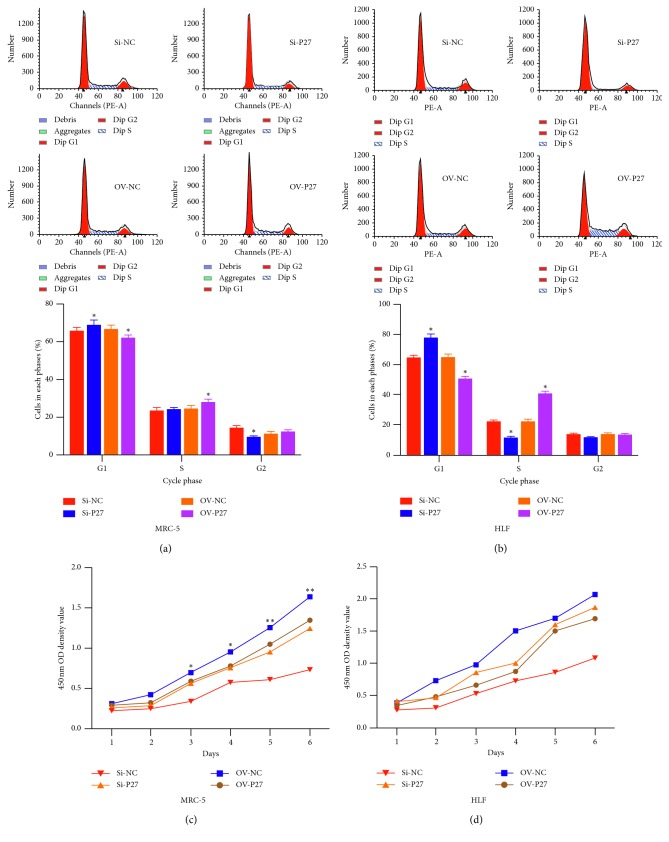
The role of P27 on the cell cycle of proliferation in MRC5 and HLF cells. (a, b) The role of P27 on the cell cycle by flow cytometry. (c, d) The role of P27 on the cell proliferation by CCK-8 assay.

To explore the role of P27 in pulmonary fibrosis, we used bleomycin to establish the mouse lung fibrosis model and examined the expression of P27 in the fibrotic mice lungs. As shown in Figure 1(a), bleomycin presented a good profibrotic role, and the expression of the lung fibrosis biomarkers a-SMA and fibronectin was significantly increased (Figures 1(b) and 1(c)). Moreover, we found that P27 expression was also upregulated in fibrotic lung tissues (Figure 2(d)).

### 3.2. Overexpression of P27 Promotes the Cell Division and Proliferation of Lung Fibroblasts

Next, we employed gain and loss of function experiments to further confirm the role of P27 in pulmonary fibrosis. Overexpression of P27 promoted the cell division of MRC5 and HLF with an increased cell number in the S stage (Figures 2(a) and 2(b)). Meanwhile, overexpression of P27 was found to promote the cell proliferation of MRC5 and HLF (Figures 2(c) and 2(d)). P27 knockdown induced the G1 stage arrest and proliferation inhibition.

### 3.3. MTORC2 Knockdown Inhibits the Expression of P27 in Human Lung Fibroblasts

**Figure 3:**
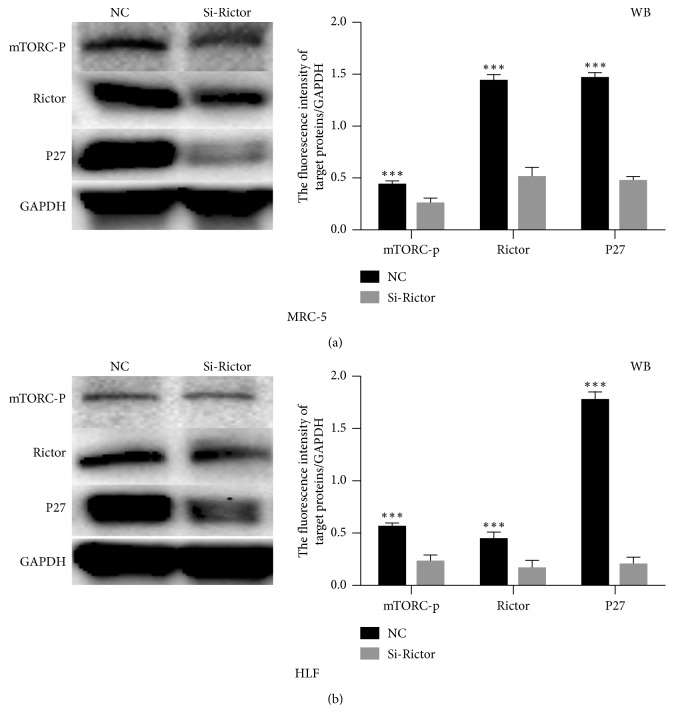
MTORC2 inhibition decreased the level of P27. (a, b) Western blot analysis of expression of mTORC-p, RICTOR, and P27 in MRC5 and HLF cells transfected with RICTOR siRNA.

To verify the role of MTORC2 in pulmonary fibrosis, we employed Rictor siRNA to disrupt the function of MTORC2. Our results showed that after Rictor knockdown, the level of phosphorylated MTORC2 was decreased and the expression of P27 was inhibited (Figure 3).

### 3.4. P27 Promotes TGF-*β*-Mediated Pulmonary Fibrosis and Is Regulated by MTORC2

**Figure 4:**
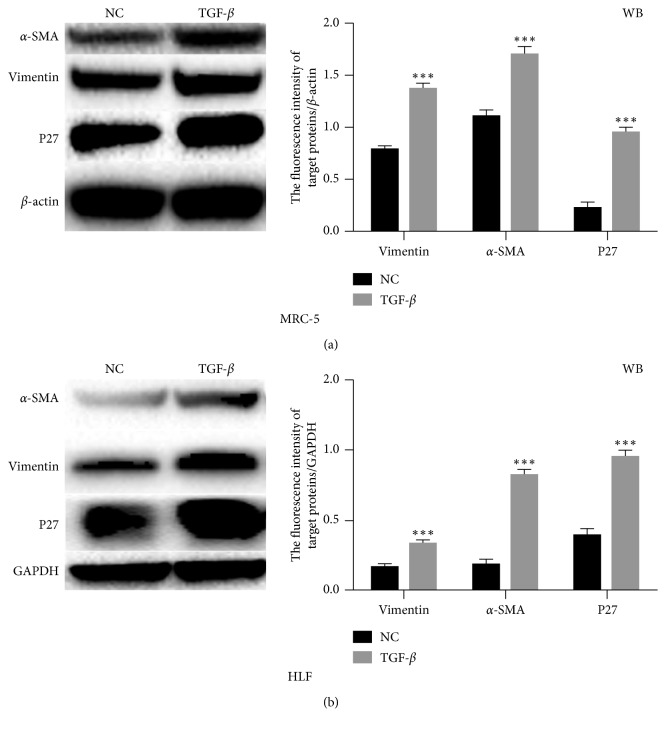
TGF-*β* promoted the expression of P27. (a, b) Western blot analysis of expression of a-SAM, vimentin, and P27 in MRC5 and HLF cells treated with TGF-*β*.

Previous study showed that TGF-*β* plays a vital role in pulmonary fibrosis via the EMT pathway [19, 20]. To confirm this, we treated MRC5 and HLF cells with TGF-*β* in the culture medium. As shown in Figure 4, TGF-*β* enhanced differentiation from fibroblast to myofibroblast proved by the upregulation of a-SMA. The EMT pathway protein vimentin was also found to be upregulated after TGF-*β* treatment, which was consistent with the previous study. Moreover, we found that TGF-*β* could enhance the level of P27, indicating that P27 was involved in TGF-*β*-induced pulmonary fibrosis.

**Figure 5:**
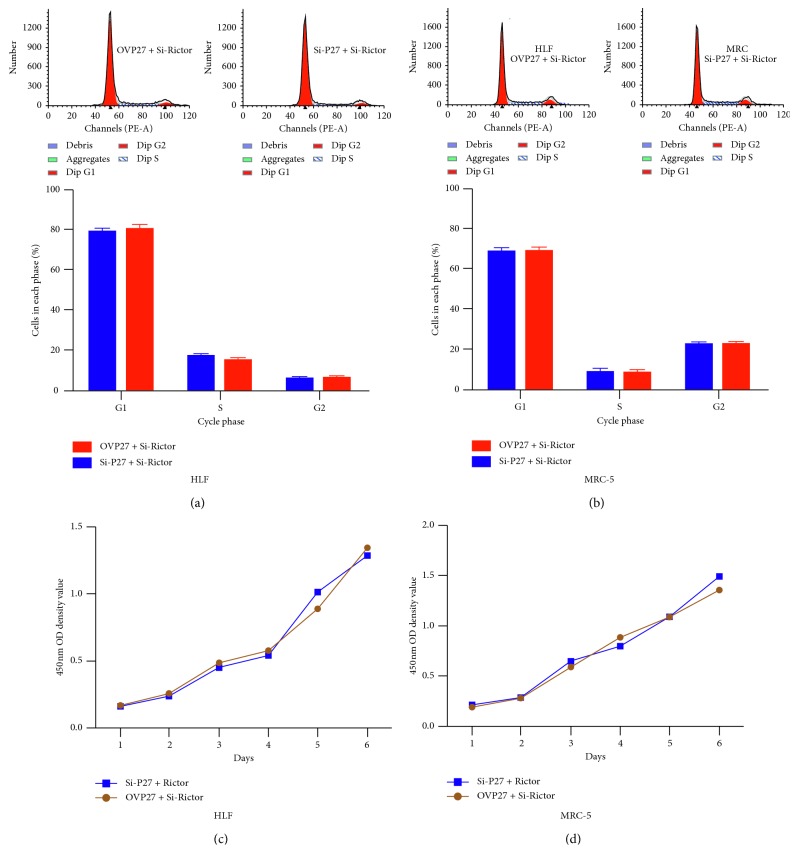
Rictor knockdown reversed the role of P27 on cell cycle and proliferation. (a, b) The cell cycle assay in MRC5 and HLF cells transfected with P27 siRNA plus Rictor siRNA or P27 ovexpression vector plus Rictor siRNA. (c, d) The cell proliferation assay in MRC5 and HLF cells transfected with P27 siRNA plus Rictor siRNA or P27 ovexpression vector plus Rictor siRNA.

Finally, we used rescue experiment to verify the relationship between MTORC2 and P27 in TGF-*β*-induced pulmonary fibrosis. There was no difference in the cell cycle and proliferation of MRC5 and HLF5 transfected with P27 siRNA plus Rictor siRNA or P27 ovexpression vector plus Rictor siRNA (Figure 5), which revealed that the role of P27 in TGF-*β*-induced pulmonary fibrosis was controlled by MTORC2.

## 4. Discussion

Pulmonary fibrosis is a chronic and progressive lung disease, in which repeated wound and repair processes lead to irreversible destruction of lung architecture [21]. The molecular mechanisms underlying pulmonary fibrosis are not well elucidated [22]. Although the exact molecular biological mechanisms of pulmonary fibrosis development are not yet clear, a growing body of researches demonstrate that TGF-*β* is a key player in fibrotic processes via inducing myofibroblast differentiation and inhibiting alveolar epithelial cell growth and repair [23, 24]. However, the detailed mechanisms of TGF-*β* underlying pulmonary fibrosis are still not clear.

In the present study, we aimed to explore the mechanisms of TGF-*β* in pulmonary fibrosis. We found that TGF-*β* could promote the EMT process via upregulating the level of vimentin in human lung fibroblasts. Some transcriptional factors such as Snail and Twist could activate the EMT process; accumulated evidences demonstrated that EMT may be a major source of pathogenic myofibroblasts during pulmonary fibrogenesis [20]. And EMT activation was previously reported to induce the expression of contractile protein *α*-SMA during IPF [25, 26]. A complicated relationship is likely to exist among lung injury, chronic inflammatory, and EMT. TGF-*β* was reported as a key regulator of the EMT process during pulmonary fibrosis [27, 28]. And in an experimental model of pulmonary fibrosis, EMT helped to activate collagen-producing fibroblasts under the regulation of TGF-*β* [29]. These results were consistent with our findings.

P27 is a member of CDK inhibitors (CKI) contributing to the cell cycle arrest in the G1 phase [30]. The role of P27 in pulmonary fibrosis in previous reports was inconsistent. Some studies showed that P27 expression in IPF tissues was ectopic, and FoxO3a could increase P27 expression to regulate IPF [6, 31, 32]. Another study demonstrated that some chemical compounds such as i-mimosine and shikonin could inhibit pulmonary fibrosis by upregulating the P27 level [33, 34]. Moreover, the interaction of P27 and TGF-*β* in pulmonary fibrosis was unknown. In the present study, we found P27 could promote pulmonary fibrosis, and TGF-*β* could upregulate the level of P27 in lung fibroblasts. These findings have not been reported before.

mTOR is a mammalian target of rapamycin (mTOR) and an important serine-threonine protein kinase of the PI3K family. It can regulate the proliferation, survival, invasion, and metastasis of tumor cells by activating ribosomal kinase [35, 36]. There are two different mTOR complexes: mTORC1 and mTORC2 [37]. mTORC2 is constituted by several components, including the rapamycin-insensitive companion of mTOR (RICTOR), DEPTOR, mLST8/G*β*L, mammalian stress-activated protein kinase-interacting protein 1 (mSIN1), Protor 1/2, TTI1, and TEL2 [15]. One study showed that TGF-*β* could induce the expression of Rictor in IPF pulmonary fibroblasts and subsequently activate mTORC2 signaling and Akt [18]. Previous studies reported that mTORC2 could regulate the expression of P27 in RCC cells [38]. However, the regulation of MTORC2 on P27 in pulmonary fibrosis was not reported previously. In this research, we, for the first time, demonstrated that mTORC2 knockdown could inhibit P27 expression in lung fibroblasts.

## 5. Conclusions

In conclusion, our results indicate that upregulated P27 participated in TGF-*β*-mediated pulmonary fibrosis, and the expression of P27 in pulmonary fibrosis tissues was regulated by MTORC2. These findings provided a novel insight into the development of pulmonary fibrosis during the progression of ILD, including IPF.

## Data Availability

The data used to support the findings of this study are available from the corresponding author upon request.
